# The Effects of Employment Conditions on Smoking Status and Smoking Intensity: The Analysis of Korean Labor & Income Panel 8^th^–10^th^ Wave

**DOI:** 10.1371/journal.pone.0057109

**Published:** 2013-02-20

**Authors:** Youn Jung, Juhwan Oh, Soonim Huh, Ichiro Kawachi

**Affiliations:** 1 School of Public Health, Seoul National University, Seoul, Republic of Korea; 2 Institute of Health Policy and Management, Medical Research Center, Seoul National University, Seoul, Republic of Korea; 3 Department of Public Administration, University of Seoul, Seoul, Republic of Korea; 4 Department of Social and Behavioral Science, Harvard School of Public Health, Boston, Massachusetts, United States of America; The University of Auckland, New Zealand

## Abstract

**Background:**

The neoliberal policies and its socioeconomic consequences in Korea have made employment conditions insecure and affected employees' health as well.

**Methods and Findings:**

To examine the association between employment condition and smoking status, we selected male respondents aged 20–59 that participated in all of the 8^th^–10^th^ wave of Korean Labor and Income Panel Study(KLIPS) which is a nationally representative data. Precarious working was significantly associated with smoking compared to standard working even after adjusting for socioeconomic indicators and self rated health status. After controlling for overall life satisfaction, the odds ratio of smoking among precarious workers decreased, but it was still marginally significant (OR = 1.43, 95% CI = 0.99 to 2.07). A relation between precarious working and heavy smoking was also significant. Precarious working was associated with a decreased likelihood of quitting smoking, while it was not significant any more after adjusting for overall satisfaction on life. Precarious work was also related to a higher likelihood of relapse among former smokers, but was not significant after adjusting for other confounders.

**Conclusions:**

Precarious workers were more likely to be smokers and heavy smokers than standard workers. Unemployment is also a significant risk factor for decreased quitting and smoking relapse. However, insecure employment was an even more consistent determinant of current smoking behavior than unemployment.

## Introduction

As a result of the rise of neoliberal policies and socioeconomic changes, employment conditions have been insecure all over the world. The standard employment contract, characterized by full-time permanent employment and regular pay, has been increasingly replaced by nonstandard forms of employment such as temporary employment [1]. Korea is not an exception. After the economic crisis of 1997 hit Korea, the Korean government was required to implement policies to promote labor market flexibility. Efforts to increase labor market flexibility resulted in the expansion of non-standard and non-regular workers accompanied by substantial lay-offs.

Insecurity in employment is linked to economic hardship as well as disadvantages in working conditions such as low and limited access to various kinds of welfare benefits. Insecure employment is also a risk factor for poor health [2], [3], [4], [5], [6], [7], [8], [9], [10], [11], [12], [13]. Kivimaki et al. reported that temporary employment was associated with increased deaths from alcohol-related causes and smoking related cancer [8]. Virtanen et al. concluded that precarious employment was positively related to anger, depression, suicide, and substance abuse [13]. Mental health or self-rated health status was also reported to be associated with insecure employment by many studies [2], [5], [6], [7], [9], [10].

However, studies on the relation between job insecurity and health behavior show more conflicting results according to the kind of health behavior [14], [15]. Based on a sample of Turkish health care workers, Cuyper ND et al. showed a positive association between temporary workers and alcohol dependence, but no significant differences were established for smoking [14]. Virtanen et al. reported a five-year study that examined changes in health behavior following the change in employment using the Health and Social Support Study in Finland. Those who were exposed to chronic unemployment and experienced a downward employment trajectory increased alcohol drinking, gained weight, and decreased physical activity and sleep duration, but smoking was not associated with employment trajectory [15]. Evidences that explain these inconsistencies remain limited. Furthermore, the associations between employment condition and health behavior such as smoking have rarely been conducted in Asian countries.

According to OECD Health Statistics 2012, the percent of adult males who are daily smokers is the highest in Korea among 33 OECD countries, which is 44.3% as of 2009 [16]. Even though the smoking rate has been decreasing due to various kinds of anti-smoking policy that Korean government had implemented during last two decades, it is still very challenging health issue in Korea.

In this study, we sought to examine the association between employment condition and health behavior, specifically smoking status by using a representative sample of Korea. For the purpose of this study, we examined whether insecure employment is associated with smoking status and smoking intensity. In addition, we examined the relation between employment status and the behavioral change in smoking

## Methods

### Study population

Data were drawn from the 8^th^, 9^th^ and 10^th^ wave of the Korean Labor and Income Panel Study (KLIPS), which include questions about smoking status. The survey passed an ethical review process by the Statistics Korea, a central government organization for statistics. This study was not required an ethical review as the KLIPS dataset was publicly opened and lack of information for individual identification. The KLIPS is a longitudinal study of a representative sample of Korean households and individuals living in urban areas. It was initiated in 1998 and is conducted annually to track the characteristics of households as well as economic activities, labor movement, income, expenditures, education, job training, and social activities of individuals [17]. The original sample of the KLIPS consisted of 5,000 households, which were sampled by two-stage stratified clustering, first selection of the enumeration districts and then selection of the households.

We only included male respondents that participated in all the 8^th^–10^th^ wave of KLIPS and whose employment conditions are permanent employees, or precarious employees or the unemployed. Our sample is limited to those aged 20–59 since the rate of retirement is high in those aged 60 or older and the relation between retirement and health is beyond the scope of this study. Final group size used in the study thus included 1,877.

### Measures

#### Employment conditions

In this study, we defined full-time, permanent employees as standard workers, and temporary, daily, part-time workers or workers with non permanent contract as precarious workers. If respondents are not currently working but they were looking for a job during the previous 4 weeks and able to work, they were defined as unemployed. We also included discouraged workers among the unemployed, i.e. those who are not seeking a job but have the intention to get a job. This is particularly important in Korea, because the proportion of discouraged workers is rapidly growing due to economic crisis but the formal unemployment rate fails to capture this population.

#### Smoking

Smoking was measured by the following questions: “Do you smoke or have you previously smoked? “ “If you are currently smoking, how many cigarettes do you smoke a day on average?” We defined current smokers as “smoker”, those who are previous smokers or never smoked as “non smoker”. Smoking intensity was categorized as (1) 40 or more cigarettes, (2) 20–39 cigarettes, (3) 10–19 cigarettes, or (4) 1–9 cigarettes per day. In this study, we classified (1) or (2) as a heavy smoker, and (3) or (4) as a light smoker.

#### Potential confounders and mediators

The following covariates were included: age, education, equivalized monthly household income, marital status, self-rated health status, overall life satisfaction. Age was categorized as four age groups (20–29, 30–39, 40–49, 50–59), and education levels were classified as middle school or less, high school, and college or more. Marital status was divided into married, single, and widowed/divorced/separated. The equivalized household income ( = total household income/family size^1/2^) was grouped into tertiles. Self-rated health status was determined by responses to the question, “How would you rate your health status?” From the five answers (very good, good, moderate, poor, very poor), a dichotomous response variable (0 = very good, good; 1 = moderate, poor, very poor) was created. Overall life satisfaction was measured by responses to the question, “How much are you satisfied with your overall life?: very satisfied, satisfied, moderate, dissatisfied, very dissatisfied” The responses were divided into a binary variable (0 = very satisfied, satisfied; 1 = moderate, dissatisfied, very dissatisfied).

#### Statistical methods

Based on the results of previous studies that factors determining smoking status and smoking intensity are different, two-part model was used in this study to analyze the independent effects of employment condition on smoking status and smoking intensity.

Using panel-logistic regression, we estimated the odds ratios(OR) and their 95% confidence intervals (CI) of current smoking and heavy smoking according to employment conditions (non-precarious vs. precarious vs. unemployed) after adjusting for age, education level, marital status, household income, self-rated health status, overall life satisfaction, and survey year.

Then the changes in smoking status during 2005–2006 and 2006–2007 were examined using logistic regression models. Odds ratios for quitting smoking among current former smokers and for smoking (re-smoking or initiating smoking) among currently former non smokers were calculated by employment conditions. We examined the effect of employment condition on smoking, firstly controlling for potential confounders such as education, marital status and self-rated health status, household income and then also controlling for potential mediators- overall satisfaction on life. The analyses were performed by STATA ver. 10.

## Results

As shown in Table 1, the proportion of single persons aged 20–29 was higher among the unemployed than among employees, although it decreased by year. Precarious workers appeared to have less education compared to standard workers and the unemployed. In 2005, 54.7% of standard workers and 43.0% of the unemployed had education level of college or higher, while only 29.0% of precarious workers were in college or higher. The proportion of married persons was lowest among the unemployed. Standard workers reported better health status compared to other groups. Overall, precarious workers and the unemployed tended to have less household income than standard workers. The proportion of persons that reported to be satisfied with their overall life was highest among standard workers (42.0% in 2005), while it was the lowest among the unemployed (14.1% in 2005). The proportion of current smokers was highest among precarious workers in 2005 and 2007, but it was highest among the unemployed group in 2006. The proportion of heavy smokers (20 or more cigarettes per day) was highest among precarious workers.

**Table 1:** Characteristics of the study sample (%).

	2005				2006				2007			
	Standard	Precarious	Unemployed	p	Standard	Precarious	Unemployed	p	Standard	Precarious	Unemployed	p
	(n = 1373)	(n = 376)	(n = 128)		(n = 1435)	(n = 378)	(n = 64)		(n = 1425)	(n = 383)	(n = 69)	
**Age group(y)**												
**20–29**	16.0	14.9	39.8	P<0.001	13.5	14.3	31.3	P<0.001	9.8	9.7	24.6	P<0.001
**30–39**	43.3	28.7	32.8		43.8	25.9	28.1		44.6	26.9	37.7	
**40–49**	27.6	32.218	20.3		27.9	31.8	25.0		29.4	31.1	18.8	
**50–59**	13.0	24.2	7.0		14.8	28.0	15.6		16.2	32.4	18.8	
**Education**												
**Mid**	8.2	26.9	14.8	P<0.001	7.7	28.6	17.2	P<0.001	7.4	29.5	15.9	P<0.001
**High**	37.1	44.2	42.2		36.8	43.9	45.3		36.1	44.7	52.2	
**Col, more**	54.7	29.0	43.0		55.5	27.5	37.5		56.5	25.9	31.9	
**Marital**												
**Single**	21.2	22.6	60.2	P<0.001	21.5	20.4	53.1	P<0.001	20.4	19.3	44.9	P<0.001
**Wid/Div/Sep**	3.1	7.5	3.1		3.1	9.0	3.1		2.8	8.4	11.6	
**Married**	75.7	67.0	36.7		75.5	70.6	43.8		76.8	72.3	43.5	
**Self-reported health**												
**Poor**	31.1	46.0	39.1	P<0.001	28.4	43.4	45.3	0.003	28.6	44.4	31.9	P<0.001
**Good**	68.9	54.0	60.9		71.6	56.6	54.7		71.4	55.6	68.1	
**Equivalized household income**												
**T1**	22.1	54.0	53.1	P<0.001	23.7	47.6	57.8	P<0.001	21.8	49.1	59.4	P<0.001
**T2**	37.1	27.1	29.7		35.6	35.2	29.7		36.7	33.4	30.4	
**T3**	40.8	18.9	17.2		40.7	17.2	12.5		41.5	17.5	10.1	
**Overall satisfaction on life**												
**Dissatisfied**	580.0	77.1	84.4	P<0.001	54.3	76.2	90.6	P<0.001	49.5	74.2	79.7	P<0.001
**Satisfied**	42.0	21.8	14.1		45.0	22.8	9.4		48.9	25.3	18.8	
**Missing**	0.1	1.1	1.6		0.7	1.1	0		1.5	0.5	1.5	
**Current smoking**												
**Yes**	55.7	67.6	57.8	P<0.001	56.0	68.0	76.6	P<0.001	53.3	69.2	68.1	P<0.001
**No**	44.3	32.5	42.2		44.0	32.0	23.4		46.7	30.8	31.9	
**Smoking intensity**												
**Heavy smoking**	15.7	26.6	18.0	P<0.001	17.2	27.0	20.3	P<0.001	15.9	27.2	20.3	P<0.001
**Light smoking**	40.1	41.0	39.8		38.8	41.0	56.3		37.3	42.0	47.8	
**Missing**	44.3	32.5	42.2		44.0	32.0	23.4		46.7	30.8	31.9	

Table 2 shows the results from panel logistic regression analyses on the employment status and current smoking. Precarious working was significantly associated with smoking compared to standard working after adjusting for socioeconomic indicators and self rated health status (OR = 1.45, 95% CI = 1.01 to 2.09, p<0.05). After controlling for overall satisfaction on life, the odds ratio of smoking among precarious workers decreased (1.45 to 1.43), but it was still marginally significant (OR = 1.43, 95% CI = 0.99 to 2.07, p<0.1). For the unemployed, the odds of being a smoker was higher compared to standard workers, but was not statistically significant. Younger age, no partner and low socioeconomic positions (less education, lower income) were strongly associated with current smoking status. Dissatisfaction on overall life also increased the odds of smoking significantly (OR = 1.50, p<0.01). Year-specific effects were not observed.

**Table 2:** Adjusted odds ratios^a^(95% confidence interval) of current cigarette smoking according to employment status.

	Model 1	Model 2	Model 3
**Age group**			
**20–29**	1.97**(1.08–3.59)	2.90***(1.44–5.86)	3.05***(1.51–6.17)
**30–39**	1.63*(0.99–2.69)	2.73***(1.58–4.72)	2.78***(1.61–4.83)
**40–49**	1.32(0.81–2.15)	1.68**(1.03–2.76)	1.63*(1.00–2.68)
**50–59**	1	1	1
**Employment condition**			
**Unemployed**	1.44(0.84–2.47)	1.13(0.66–1.96)	1.01(.058–1.76)
**Precarious**	1.99***(1.39–2.85)	1.45**(1.01–2.09)	1.43*(0.99–2.07)
**Standard**	1	1	1
**Education level**			
**Middle school**		7.84***(3.96–15.5)	7.31***(3.808–14.53)
**High school**		3.34***(2.20–5.11)	3.11***(2.03–4.77)
**College, more**		1	1
**Marital status**			
**Single**		1.75**(1.08–2.84)	1.62*(0.99–2.63)
**Widowed, divorced, separated**		5.09***(2.05–12.63)	4.69***(1.90–11.68)
**Married**		1	1
**Self-reported health status**			
**Poor**		0.92(0.73–1.17)	0.88(0.69–1.13)
**Good**		1	1
**Equivalized household income**			
**T1**		1.54**(1.09–2.19)	1.49**(1.04–2.12)
**T2**		1.21(0.90–1.63)	1.18(0.88–1.59)
**T3**		1	1
**Overall satisfaction on life**			
**Dissatisfied**			1.50***(1.18–1.91)
**Satisfied**			1
**Survey Year**			
**2005**		1.04(0.84–1.28)	1.00(0.80–1.23)
**2006**		1.19* (0.97–1.47)	1.15(0.93–1.42)
**2007**		1	1

* : p<0.1.

** : p<0.05,

*** : p<0.01.

Table 3 shows adjusted ORs (95% CI) of heavy smoking according to employment status. There was a relation between precarious working and heavy smoking. Even after adjusting for socioeconomic indicators, self rated health status and overall satisfaction on life, these associations were still strongly significant (OR = 1.48, p<0.01). Education levels less than college were significantly associated with an increased likelihood of smoking 20 or more cigarettes per day (p<0.01).

**Table 3:** Adjusted odds ratios(95% confidence interval) of heavy smoking according to employment status.

	Model 1	Model 2	Model 3
**Age group**			
**20–29**	0.37***(0.23–0.58)	0.56**(0.32–0.97)	0.59*(0.34–1.02)
**30–39**	0.66**(0.46–0.95)	0.89(0.59–1.34)	0.89(0.59–1.34)
**40–49**	0.74(0.51–1.07)	0.82(0.56–1.20)	0.80(0.55–1.17)
**50–59**	1	1	1
**Employment condition**			
**Unemployed**	1.28(0.78–2.09)	1.19(0.72–1.97)	1.17(0.71–1.94)
**Precarious**	1.66***(1.26–2.19)	1.48***(1.10–1.97)	1.48***(1.11–1.98)
**Standard**	1	1	1
**Education level**			
**Middle school**		2.03***(1.30–3.16)	2.02***(1.29–3.15)
**High school**		2.07***(1.54–2.79)	2.07***(1.54–2.79)
**College, more**		1	1
**Marital status**			
**Single**		0.83(0.58–1.18)	0.80(0.56–1.15)
**Widowed, divorced, separated**		1.25(0.73–2.15)	1.25(0.73–2.16)
**Married**		1	1
**Self-reported health status**			
**Poor**		1.05(0.84–1.31)	1.06(0.84–1.33)
**Good**		1	1
**Equivalized household income**			
**T1**		0.92(0.68–1.26)	0.94(0.68–1.29)
**T2**		1.04(0.78–1.38)	1.04(0.78–1.39)
**T3**		1	1
**Overall satisfaction on life**			
**Dissatisfied**			1.05(0.82–1.33)
**Satisfied**			1
**Survey Year**			
**2005**		0.97(0.77–1.21)	0.98(0.78–1.23)
**2006**		1.07(0.86–1.33)	1.07(0.85–1.33)
**2007**		1	1

* : p<0.1.

** : p<0.05,

*** : p<0.01.

Table 4 describes the results from binary logistic regression analyses on the associations between employment status and quitting smoking among 2005 or 2006 smokers. Precarious working was significantly associated with a decreased likelihood of quitting smoking (OR = 0.77, 95% CI = 0.59 to 1.02, p<0.1 in model 2), while it was not significant any more after adjusting for overall satisfaction on life. Lower education was related to a lower likelihood of quitting smoking (for middle school, OR = 0.61, p<0.01; for high school, OR = 0.75, p<0.01). The likelihood of quitting smoking among widowed, divorced, and separated was significantly low compared to married persons (OR = 0.56, p<0.1).

**Table 4:** Adjusted odds ratios^a^ (95% confidence interval) of quitting smoking according to employment status among 2005 or 2006 smokers (N = 2,604).

	Model 1	Model 2	Model 3
**Employment condition**			
**Unemployed**	0.75(0.46–1.24)	0.84(0.51–1.41)	0.90(0.54–1.50)
**Precarious**	0.67***(0.51–0.88)	0.77*(0.59–1.02)	0.80(0.60–1.07)
**Standard**	1	1	1
**Education level**			
**Middle school**		0.59**(0.40–0.90)	0.61**(0.40–0.92)
**High school**		0.72***(0.57–0.91)	0.75**(0.59–0.95)
**College, more**		1	1
**Marital status**			
**Single**		1.14(0.86–1.51)	1.16(0.86–1.55)
**Widowed, divorced, separated**		0.53**(0.29–0.96)	0.56*(0.31–1.00)
**Married**		1	1
**Self-reported health status**			
**Poor**		0.81* (0.64–1.02)	0.82(0.64–1.05)
**Good**		1	1
**Equivalized household income**			
**T1**		0.99(0.74–1.31)	1.00(0.75–1.34)
**T2**		0.92(0.71–1.20)	0.91(0.70–1.19)
**T3**		1	1
**Overall satisfaction on life**			
**Dissatisfied**			0.85(0.67–1.08)
**Satisfied**			1

* : p<0.1.

** : p<0.05,

*** : p<0.01.

a adjusted for age.

Table 5 shows the adjusted odds ratios of re-initiating smoking according to employment status among 2005 or 2006 non-smokers. The likelihood of re-initiating smoking among the unemployed was significantly high after adjustment for socioeconomic position, health status (OR = 1.80, 95% CI = 1.07 to 3.03, p<0.05). Precarious work was also related to a higher likelihood of re-initiating smoking, but was not significant after adjusting for other confounders. Lower education and low income level was associated with re-initiating smoking. Overall life satisfaction significantly decreased the likelihood of re-initiating smoking (OR = 1.60, p<0.01).

**Table 5:** Adjusted odds ratios^a^(95% confidence interval) of re-initiating smoking according to employment status among 2005 or 2006 non-smokers (N = 1,797).

	Model 1	Model 2	Model 3
**Employment condition**			
**Unemployed**	2.52***(1.55–4.10)	1.80**(1.07–3.03)	1.66*(0.99–2.80)
**Precarious**	1.51***(1.12–2.03)	1.09(0.79–1.49)	1.05(0.76–1.45)
**Standard**	1	1	1
**Education level**			
**Middle school**		2.71***(1.73–4.25)	2.53***(1.61–3.98)
**High school**		1.65***(1.25–2.17)	1.52***(1.14–2.01)
**College, more**		1	1
**Marital status**			
**Single**		1.22(0.88–1.70)	1.12(0.80–1.58)
**Widowed, divorced, separated**		2.06**(1.05–4.03)	1.79*(0.93–3.52)
**Married**		1	1
**Self-reported health status**			
**Poor**		1.02(0.79–1.31)	0.92(0.71–1.20)
**Good**		1	1
**Equivalized household income**			
**T1**		1.64***(1.20–2.23)	1.47**(1.07–2.03)
**T2**		0.97(0.72–1.30)	0.87(0.65–1.18)
**T3**		1	1
**Overall satisfaction on life**			
**Dissatisfied**			1.60***(1.23–2.10)
**Satisfied**			1

* : p<0.1.

** : p<0.05,

*** : p<0.01.

a adjusted for age.

## Discussion

Our findings show that marginalization in the labor market is associated with likelihood of being a smoker as well as a heavy smoker. After the effects of age, education, marital status, income and self-reported health status were accounted for, precarious workers were more likely to be smokers than standard workers. Among smokers, a higher likelihood of being a heavy smoker was significantly associated with precarious work. Moreover, precarious workers were less likely to quit smoking. This is in contrast to a previous Turkish study reporting that there was no association between temporary employment and smoking status [14]. Differences in social context and measurement for smoking status could have contributed to the inconsistent findings. Furthermore, the Turkish study targeted some health care workers, i.e. non-population-based samples. We also found that unemployment increased the likelihood to be a smoker and a heavy smoker compared to standard work, despite these associations being not significant. Small sample sizes of the unemployed may possibly have decreased the likelihood of detecting significant associations. However, unemployment was strongly associated with re-initiating smoking in our results. These results are consistent with earlier studies reporting an association of unemployment with smoking [18], [19], [20].

After the economic crisis of 1997, Korea has experienced a full-scale restructuring of its labor market, including massive layoffs and flexible contracts. This insecure labor market condition might give rise to psychological distress, which can lead to unhealthy behaviors such as smoking, alcohol consumption, etc, to both the insecurely employed as well as the unemployed. In addition, unstable job position, unstable life and less favorable working conditions in precarious work (such as low salaries, limited access to welfare benefits and less job control) may prevent such workers from quitting smoking partly due to the lack of coping resources to manage their stress. These hypotheses are supported by some evidence that work stress is associated with smoking [21].

Risky lifestyle contributes to poor health and excess mortality among the temporary workers and the unemployed [8], [22]. Given the Korean context, where the smoking rate in adults is very high and the prevalence of smoking-related cancer is growing, our findings have important public health implications. Lifestyles such as smoking may be viewed as a matter of individual free choice on the one hand, but there also exist strong structural determinants in our society that limit the choice to quit or resist smoking [15]. Our findings suggest that there is a need to implement more active policies to address the fundamental cause of risky health behavior: In addition to individual level health policies, for example, increasing the opportunity for health promotion, there is a need for structural policies that reduce the economic and psychological distress of the unemployed and the precarious workers.

This study is the first population-based longitudinal study to examine the association between employment condition and smoking in Korea. However, there are several limitations in our study. First, our study included only male respondents. The reason for excluding female respondents from this study was the small sample size partly due to women's tendency to underreport their smoking status. In the Korean context, smoking is viewed as a taboo for women which makes it difficult to obtain accurate estimates.. Given the conditions that female smoking rate is rapidly increasing and Korean women are more marginalized in the labor market than men, future research is needed to investigate the relationship between employment condition and smoking in women. Second, our results are not perfectly free from omitted variable biases because we used random effects panel model instead of using fixed effects panel model. The presence of unobserved common determinants of smoking and employment status can lead to biased estimation. Fixed-effect estimation provides the strongest control over the confounding influences of unobserved individual-specific effects, but it cannot identify the effects of individual time-stable explanatory variables that are interesting parameters in our study, such as education level. Furthermore, no difference in smoking status was observed in most individuals, which may be related to the short follow-up period (and would have led to a large reduction in the effective sample size had we attempted a fixed effects analysis). Instead, we used a random-effects model that assumes that individual-specific effects are randomly drawn from some well-defined probability distribution.

Despite these limitations, our findings suggest a consistent relationship between precarious employment status and smoking among males. Specifically, by showing that precarious employment status was associated with both current smoking status as well as changes in smoking habit (quitting and relapse), we could partly exclude the possibility of reverse causality between employment status and smoking (cigarette smoking might have caused people to become marginalized in labor market). These findings emphasize the need to implement active policies to prevent the adverse health consequences of marginalization in the labor force.
